# Electrophysiological and behavioral characterization of bioactive compounds of the *Thymus vulgaris*, *Cymbopogon winterianus*, *Cuminum cyminum* and *Cinnamomum zeylanicum* essential oils against *Anopheles gambiae* and prospects for their use as bednet treatments

**DOI:** 10.1186/s13071-015-0934-y

**Published:** 2015-06-11

**Authors:** Emilie Deletre, Fabrice Chandre, Livy Williams, Claire Duménil, Chantal Menut, Thibaud Martin

**Affiliations:** UR Hortsys, Cirad, Campus de Baillarguet, 34980 Montferrier, France; UMR MiVEGEC, IRD-CNRS-UM1-UM2, 911 Ave Agropolis, 34980 Monferrier, France; USDA-ARS, European Biological Control Laboratory, Campus de Baillarguet, 34980 Monferrier, France; Institut des Biomolécules Max Mousseron, Faculté de Pharmacie, 15 Av.Charles Flahault, 34000 Montpellier, France; Plant Health Department, ICIPE, P.O. Box 30772-00100, Nairobi, Kenya

**Keywords:** Essential oil, Repellency, Toxicity, Vector control, DEET, Permethrin

## Abstract

**Background:**

Laboratory and field studies showed that repellent, irritant and toxic actions of common public health insecticides reduce human-vector contact and thereby interrupt disease transmission. One of the more effective strategies to reduce disease risk involves the use of long-lasting treated bednets. However, development of insecticide resistance in mosquito populations makes it imperative to find alternatives to these insecticides. Our previous study identified four essential oils as alternatives to pyrethroids: *Thymus vulgaris*, *Cymbopogon winterianus*, *Cuminum cyminum*, *Cinnamomum zeylanicum*. The objectives of this study were to identify active compounds of these essential oils, to characterize their biological activity, and to examine their potential as a treatment for bednets.

**Methods:**

We evaluated the electrophysiological, behavioural (repellency, irritancy) and toxic effects of the major compounds of these oils against *Anopheles gambiae* strain ‘Kisumu’.

**Results:**

Aldehydes elicited the strongest responses and monoterpenes the weakest responses in electroantennogram (EAG) trials. However, EAG responses did not correlate consistently with results of behavioral assays. In behavioral and toxicity studies, several of the single compounds did exhibit repellency, irritancy or toxicity in *An. gambiae*; however, the activity of essential oils did not always correlate with activity expected from the major components. On the contrary, the biological activity of essential oils appeared complex, suggesting interactions between individual compounds and the insect under study. Data also indicated that the three effects appeared independent, suggesting that repellency mechanism(s) may differ from mechanisms of irritancy and toxicity.

**Conclusions:**

Based on the bioassays reported here, some of the compounds merit consideration as alternative bednet treatments.

## Background

*Anopheles gambiae* Giles, 1902 complex includes major vectors responsible for the transmission of *Plasmodium* spp., particularly *Plasmodium falciparum*, which is the most hazardous protozoan parasite that causes malaria infection in humans [1]. One strategy to reduce vector transmission of pathogens that cause malaria is the through strategies which involve protection against mosquito bites. Bednets treated with pyrethroids act as a physicochemical barrier and thereby disrupt the vector-host contact. Pyrethroids are used because they are relatively safe for humans and they have rapid excito-repellent, knock-down and killing effects [2]. However, pyrethroid resistance has been reported in 27 countries from sub-Saharan Africa, underscoring the urgent need to find alternatives to these insecticides [3–5].

Plant-produced compounds have demonstrated efficacy in the prevention of mosquito bites [6]. Some of the better known repellents are citronellal, myrcene, geraniol, citral, limonene, pinenes, citronellol, eugenol, and linalool [6]. These natural compounds are biodegradable and environmentally friendly and are well accepted by people who want to avoid synthetic chemicals [7]. Terpenoids are the major constituents of essential oils. Essential oils are blends comprised of 30 to 100 different compounds (or more according to their source) in various proportions. From Ipek *et al.* [8], two or three of the major compounds of an essential oil are usually responsible for their biological activity. With multiple bioactive compounds present in an essential oil, the oil can affect multiple targets at the same time; therefore, neither resistance nor adaptation to these products has been yet documented [9]. Despite their wide use, it is important to improve upon our knowledge of bioactive compound(s) to better understand their full potential as repellents and/or insecticides.

In previous studies we evaluated promising essential oils from four plants: *Thymus vulgaris*, *Cymbopogon winterianus*, *Cuminum cyminum*, *Cinnamomum zeylanicum* for their repellent, irritant and toxic effect [10]. These oils and their constituents might function as either topical repellents for use on skin or as a treatment for bednets, but their active compounds are still unidentified. The objectives of the present study were to identify bioactive compounds in these essential oils, and to evaluate the responses of *An. gambiae* mosquitoes to these compounds using electrophysiological and behavioural assays that will shed light on whether they are suitable candidates for bednet treatment.

## Methods

### Mosquitoes

Behavioural assays were performed using female *An. gambiae* originating from the insecticide susceptible reference Kisumu strain. This strain, originally collected in Kenya in 1953, has been reared at LIN-IRD, Montpellier, France. The insecticide susceptibility of the Kisumu strain was confirmed with World Health Organization (WHO) diagnostic doses (i.e. 4 % DDT, 0.75 % permethrin) and is controlled every 4 months as recommended by the ISO 9001 norm. The colony was maintained in a climatic controlled room at 27 ± 2 °C, 80 ± 10 % RH and with a photoperiod cycle of 12 h Light: 12 h Dark. Mosquito larvae were fed a diet of fish food (TetraMin). Emerged adults were mechanically aspirated and transferred into 25 × 25 × 25 cm cages and provided access to 10 % honey-water solution.

### Products

Studies were performed with four plant essential oils: citronella (leaf), *Cymbopogon winterianus* (Nactis, France, lot 40018500); cumin (seed), *Cuminum cyminum* (Ipra, France, lot 902560); cinnamon (bark), *Cinnamomum zeylanicum* (Nactis, France); and thyme (leaf), *Thymus vulgaris* (Huiles & sens, France, lot A2) and 19 chemical standards (Sigma Aldrich, St Louis, MO, USA): citronellal (≥95 % purity), geraniol (98 % purity), citronellol (≥ 95 % purity), (S)-(-)-limonene (96 % purity), geranyl acetate (98 % purity), cuminaldehyde (98 % purity), (-)-β-pinene (99 % purity), γ-terpinene (≥ 97 % purity), *p*-cymene (99 % purity), (E)-cinnamaldehyde (99 % purity), 2-methoxy-cinnamaldehyde (98 % purity), cinnamyl acetate (99 % purity), thymol (99.5 % purity), carvacrol (≥ 98 % purity), α-terpinene (85 % purity), linalool (97 % purity), and β-caryophyllene (≥ 80 % purity), and (N,N-diethyl-3-methylbenzamide (DEET) and permethrin (≥ 80 % purity) from Sigma-Aldrich, France. For the tunnel test (see below), formulated permethrin (PERIPEL 10 EC, Bayer Crop Science) was used. The pyrethroid permethrin, mainly used in mosquito nets and the insect repellent DEET, which is effective at reducing mosquito [11–13], have been used as positive controls.

Four blends were prepared, each comprised of the major compounds found within the 4 selected essential oils: citronella blend (citronellal, geraniol, citronellol, limonene and geranyl acetate), cumin blend (cuminaldehyde, β-pinene, γ-terpinene and *p*-cymene), cinnamon blend (cinnamaldehyde, 2-methoxy-cinnamaldehyde and cinnamyl acetate) and thyme blend (thymol, p-cymene, carvacrol, α-terpinene, linalool and β-caryophyllene). Each blend was prepared by diluting the major compounds in ethanol in a ratio based on their respective proportions in the essential oils. DEET, permethrin, the four essential oils, the 17 essential oils compounds and the four blends were diluted at 0.1 and 1 % (v/v for liquid compound or w/w for powdered compound) in a solvent that consisted of ethanol (2/3) and silicone oil Dow Corning 556 (1/3). All major compounds were tested at the relative concentration that they are found in the essential oils [10], at the efficient concentration (concentration C2) and 1/10 of this concentration (concentration C1) (Table 1). For instance, citronellal accounts for approximately 34.7 % of the citronella essential oil. The citronella oil was efficient at 1 %, so the citronellal was tested at C2 = 0.35 % (0.03 mg/cm^2^) and 10 times less at C1 = 0.035 % (0.003 mg/cm^2^). By diluting in this manner, the quantity of a compound tested was approximately the same within the essential oil, the blend and for the compound alone. Each assay with a treatment was preceded by evaluation of a negative control that consisted of the solvent ethanol-silicone oil. In spatial repellency assays, 3.3 mL of this solution was deposited on a 13 × 30 cm chromatography paper except on a border margin of 1.5 cm width. For contact irritancy and toxicity assays, 2 mL of the solution was deposited on 12 × 15 cm chromatography paper.

**Table 1:** Ratios and quantities of individual compounds of the essential oils: citronella, cumin, thyme and cinnamon

Essential oil and plant species	Composition (%)^*a*^		Quantity tested (μl/cm^*2*^)^*b*^	
			C1	C2
Citronella	34.7 %	Citronellal	0.004	0.035
*Cymbopogon winterianus*	22.5 %	Geraniol	0.002	0.023
	12.0 %	Citronellol	0.001	0.012
	3.5 %	Geranyl-acetate	0.0003	0.003
	3.3 %	Limonene	0.0003	0.003
	76.0 %	Sub-total (blend)	0.010	0.100
	4.2 %	Elemol	NT	NT
	2.9 %	Citronellyl acetate	NT	NT
	2.5 %	β-elemene	NT	NT
	2.2 %	δ-cadinene	NT	NT
	0.9 %	Linalool	NT	NT
	0.8 %	Eugenol	NT	NT
	89.5 %	Total	NT	NT
Cumin	30.1 %	Cuminaldehyde	0.003	0.030
*Cuminum cyminum*	12.2 %	β-pinene	0.001	0.012
	11.6 %	γ-terpinene	0.001	0.012
	9.7 %	*p*-cymene	0.001	0.097
	63.6 %	Sub-total (blend)	0.010	0.100
	16.6 %	p-mentha-1,3-dien-7-al	NT	NT
	8.8 %	p-mentha-1,4-dien-7-al	NT	NT
	0.6 %	α-pinene	NT	NT
	0.4 %	Myrcene	NT	NT
	0.4 %	Limonene	NT	NT
	90.4 %	Total	NT	NT
Thyme	30.5 %	Thymol	0.003	0.031
*Thymus vulgaris*	23.7 %	*p*-cymene	0.002	0.024
	13.6 %	Carvacrol	0.001	0.014
	8.4 %	α-terpinene	0.001	0.008
	4.0 %	Linalool	0.0004	0.004
	3.5 %	β-caryophyllene	0.0004	0.004
	83.7 %	Sub-total (blend)	0.010	0.100
	1.7 %	Myrcene	NT	NT
	1.1 %	Borneol	NT	NT
	1.1 %	α-pinene	NT	NT
	1.4 %	γ-terpinene	NT	NT
	1.2 %	Terpinen-4-ol	NT	NT
	0.9 %	Limonene	NT	NT
	0.8 %	α-thujene	NT	NT
	91,9 %	Total	NT	NT
Cinnamon	78.5 %	(E)-cinnamaldehyde	0.008	0.079
*Cinnamomum zeylanicum*	9.6 %	2-methoxy-cinnamaldehyde	0.001	0.096
	3.1 %	Cinnamyl-acetate	0.003	0.031
	91.2 %	Sub-total (blend)	0.0100	0.1000
	1.1 %	Benzaldehyde	NT	NT
	0.9 %	Coumarine	NT	NT
	0.7 %	Phenyl ethyl alcohol	NT	NT
	0.4 %	(Z)-cinnamaldehyde	NT	NT
	94,3 %	Total	NT	NT

^a^The percentage composition of the essential oil was computed by the normalization method from GC/FID analyses, response factors being taken as one for all compounds. The composition of the four essential oils was identified by gas chromatography and mass spectrometry

^b^The used quantities are expressed in μl/cm^2^ of chromatograph paper or net

**Table 2:** Residual efficacy of the net treatment on Anopheles gambiae^a^

Product	Time (h)	n	Irritated		Knocked-down		Killed	
Control	0	66	6.1	(0.3–11.9)^b^	0.0	(0.0–0.0)	1.5	(−1.4–4.4)
Geraniol	0	61	45.9	(33.4–58.4)^c^	11.5	(3.5–19.5)	16.4	(7.1–25.7)
Geraniol	3	65	38.5	(26.7–50.3)	3.1	(−1.1–7.3)	12.3	(4.3–20.3)
Geraniol	6	66	34.8	(23.3–46.3)	3.0	(−1.1–7.1)	0.0	(0.0–0.0)
Geraniol	9	60	36.7	(24.5–48.9)	0.0	(0.0–0.0)	0.0	(0.0–0.0)
Control	9	65	7.7	(1.2–14.2)	0.0	(0.0–0.0)	1.5	(–1.5–4.5)
*p*-value (model estimate)^d^			0.259		<0.001 (−0.4)		<0.001 (−0.6)	
Control	0	62	9.7	(2.3–17.1)	0.0	(0.0–0.0)	0.0	(0.0–0.0)
Cinnamaldehyde	0	62	35.5	(23.6–47.4)	11.3	(3.4–19.2)	82.3	(72.8–91.8)
Cinnamaldehyde	3	66	40.9	(29.0–52.8)	6.1	(0.3–11.9)	68.2	(57.0–79.4)
Cinnamaldehyde	6	61	54.1	(41.6–66.6)	19.7	(9.7–29.7)	60.7	(48.4–73.0)
Cinnamaldehyde	9	56	62.5	(49.8–75.2)	16.1	(6.5–25.7)	57.1	(44.1–70.1)
Control	9	62	9.7	(2.3–17.1)	0.0	(0.0–0.0)	3.2	(−1.2–7.6)
*p*-value (model estimate)			0.001 (0.1)		0.006 (−0.1)		0.003 (−0.1)	
Control	0	63	6.3	(0.3–12.3)	0.0	(0.0–0.0)	1.6	(−1.5–4.7)
Carvacrol	0	61	14.8	(5.9–23.7)	9.8	(2.3–17.3)	86.9	(78.4–95.4)
Carvacrol	3	67	43.3	(31.4–55.2)	17.9	(8.7–27.1)	64.2	(52.7–75.7)
Carvacrol	6	65	46.2	(34.1–58.3)	40.0	(28.1–51.9)	43.1	(31.1–55.1)
Carvacrol	9	54	20.4	(9.7–31.1)	22.2	(11.1–33.3)	48.1	(34.8–61.4)
Control	9	71	18.3	(9.3–27.3)	0.0	(0.0–0.0)	2.8	(−1.0–6.6)
*p*-value (model estimate)			0.368		<0.001 (−0.2)		<0.001 (−0.2)	
Control	0	65	7.7	(1.2–14.2)	0.0	(0.0–0.0)	1.5	(−1.5–4.5)
Cuminaldehyde	0	67	52.2	(40.2–64.2)	22.4	(12.4–32.4)	38.8	(27.1–50.5)
Cuminaldehyde	3	59	61.0	(48.6–73.4)	0.0	(0.0–0.0)	5.1	(−0.5–10.7)
Cuminaldehyde	6	71	42.3	(30.8–53.8)	0.0	(0.0–0.0)	12.7	(5.0–20.4)
Cuminaldehyde	9	63	25.4	(14.7–36.1)	0.0	(0.0–0.0)	1.6	(−1.5–4.7)
Control	9	64	10.9	(3.3–18.5)	0.0	(0.0–0.0)	0.0	(0.0–0.0)
*p*-value (model estimate)			<0.001 (−0.1)		1.00		<0.001 (−0.2)	

^a^Proportion of 4- to 7-day-old, non-blood-fed, sugar-fed, Kisumu strain females that were irritated, knocked down, and killed by geraniol (0.023 μl/cm^2^), cinnamaldehyde (0.079 μl/cm^2^), carvacrol (0.014 μl/cm^2^) and cuminaldehyde (0.030 μl/cm^2^) after 0, 3, 6 and 9 h of the net treatment

^b^confidence interval calculated with the Wald method

^c^Pairwise comparison of proportion was done using Fisher’s test. Values in bold lettering were significantly different from the controls with the Holm’s sequential Bonferroni correction method

^d^ *P*-value and model estimate of the generalized linear model of the time on the mosquito repellency, knock down effect, and mortality

We impregnated 100 denier multifilament polyester netting for the residual effect assays with WHO tests kits (17 cm × 20 cm) and the tunnel tests (25 cm × 25 cm), respectively. Polyester nets were impregnated with 1.9 ml and 3.5 ml solution respectively to obtain C2 (i.e. geraniol: 0.023 μl/cm^2^, cinnamaldehyde: 0.079 μl/cm^2^, carvacrol: 0.014 μl/cm^2^, and cuminaldehyde: 0.030 μl/cm^2^. The small pieces of net closing the tubes were also impregnated with the tested product. These volumes of solution corresponded to the specific absorption capacity of the net (56 ml/m^2^) previously calculated according to WHOPES procedure [14]. The nets were allowed to dry for 30 min before the first test. For the tunnel tests, we used 0.1 μl/cm^2^ each of permethrin, geraniol, cinnamaldehyde, carvacrol, cuminaldehyde, blends comprised of these last four products, citronella oil, cinnamon oil, thyme oil, cumin oil or linalool. Linalool was included in these tests because it was electrophysiologically active in EAG trials.

### Gas chromatography analysis

The four essential oils (citronella, cinnamon, cumin, and thyme) were analysed on a Varian gas chromatograph, model CP-3380, equipped with a flame ionisation detector (FID). The FID was operated at 220 °C and separation was effected using an HP_5 J&W Agilent (5-phenyl-95 % methylpolysiloxane) capillary column (30 m × 0.25 mm, film thickness 0.25 μm). Injector and detector temperatures were set at 220 and 250 °C, respectively. The oven temperature was held at 60 °C for 1 min after injection, then programmed to increase at 3 °C min^−1^ to 220 °C and held at 220 °C for 1 min. The carrier gas was N_2_ set at a flow rate of 0.8 ml/min. A 1 μl solution (10 % essential oil in ethyl ether) was injected manually. A blend of alkanes (C9-C22) was injected to calculate the retention index: RI = [TR(X)−TR(n)]/[TR(n + 1)−TR(n)]*100 + 100*n where TR(X) is the retention time of a studied product, TR(n) is the retention time of the alkane with *n* carbons that eluted before X, TR(n + 1) is the retention time of the alkane of *n* + 1 carbons that eluted after X. The percentage composition of the essential oil was computed by the normalization method from GC/FID analyses, response factors were assumed equal to one for all compounds.

### Coupled gas chromatography mass spectrometry analysis

GC-MS analyses were performed on a Hewlett Packard 5890 II gas chromatograph, interfaced to a single quadrupole mass selective detector (Model 5972). The column was a HP-5 MS capillary column (30 × 0.25 mm, film thickness 0.25 mm). Helium was the carrier gas, set at a flow rate of 0.6 ml/min. Injector and MS transfer line temperatures were set at 220 and 250 °C, respectively. The oven programme temperature was identical to that used in GC-FID analysis. Diluted samples (10:100 in CH_2_Cl_2_, v/v) of 1 μL were injected manually and in a split mode (1:100 split ratio). The MS was operated in the electron ionization (EI) mode with the filament set at 70 eV. Data were acquired over a range *m*/*z* 35-300 with a scan rate of 2.96 scan s^−1^. The electron multiplier was set to 1460 eV. The identification of the compounds was accomplished by comparison of their relative retention indices as well as comparison of mass spectra with those of standards (for main components), those found in the literature [15] and those supplemented by the NBS75K database and Wiley 7^th^ NIST 98 EPA/NIH Mass Spectral Library Upgrade (provided by Hewlett Packard with the GC/MS control and data processing software).

### Electrophysiology

The major compounds were tested individually as olfactory stimuli using an EAG system. For the EAG recording, each compound was diluted to 1 % in absolute ethanol (Carlo-Erba Reagents, Val de Reuil, France). Stimulus applicators were prepared by pipetting 25 μl of a test solution onto a 6 cm by 0.5 cm strip of Whatman No. One filter paper (Whatman International Ltd., Maidstone, Kent, UK), after which the filter paper was placed inside a 14.5-cm long glass Pasteur pipette. Fresh stimulus applicators were prepared after 2 h of use. Three controls were used: 1) an empty pipette, 2) a pipette containing 25 μl ethanol only on filter paper, and 3) a pipette containing 25 μl 100 μM octanal in ethanol on filter paper (octanal standard).

The EAG apparatus (Syntech Ltd., Hilversum, The Netherlands) was linked to a desktop computer (with IDAC-02 data acquisition interface board) on which recording, storing, and quantifying EAG responses were performed. The recording and indifferent electrodes were silver wires enclosed in drawn glass capillary tubes filled with phosphate buffered saline (NaCl, 4 g; Na_2_HPO_4_, 0.57 g; KH_2_PO_4_, 0.1 g; KCl, 0.1 g in 500 ml distilled water; pH 7.4).

Non-blood-fed females *An. gambiae* (4 to 7 days after emergence) were cooled in a refrigerator (4 °C) before excising the head with a scalpel. Both antennae remained intact and the tip of one randomly chosen antenna was removed with a scalpel. The recording electrode was placed on the tip of the cut antenna. The antennal preparation was bathed continuously by a stream of charcoal-filtered and humidified air at a flow rate of 1 l/min. Air temperature and relative humidity was measured 15 cm from the antennal preparation (overall ranges for all trials: 21–25 °C, 42–68 % RH). EAG recording began 6 min after the antennal preparation was mounted. At this time, the following test protocol was used for each recording trial. The controls were tested in the following order (empty, ethanol, octanal, empty), after which the first nine randomly chosen chemical treatments among the possible 17 were tested, then the controls again, then the last eight randomly chosen chemical treatments, and finally the controls again. Presentation of controls throughout the recording session permitted standardization of antennal responses. Test compounds and controls were applied (0.5 s pulse) at 30 s intervals separated by a purge of filtered-humidified air via an aluminum tube ca. 5 mm from the antenna. EAGs were measured as maximum amplitude of depolarization (mV). Each chemical was tested on 28 individuals.

Maximum EAG responses were control-adjusted with the ethanol only control, and expressed as proportional responses relative to the octanal standard. These data were then square root-transformed 0.5(√x + √(x + 1)) [16] and analysis of variance was used to compare maximum EAG deflection between chemicals followed by a posthoc Tukey’s HSD pairwise comparison test with the R 2.12.2software [17].

### Behavioral bioassays

Detailed descriptions of the apparatus, assay protocols, and data analysis procedures have been published previously [10]. To summarize, bioassays were conducted between 10 am and 6 pm local hours at 24 ± 1 °C and 50 ± 10 % RH, and for each product, all assays were performed the same day.

### Spatial repellency assays

The apparatus is a cylinder divided into two chambers, one treated and one untreated. Treated papers, with products or with only the solvent for control, were rolled around the inner surface of the treated chamber, whereas the inner surface of the untreated chamber was covered by untreated chromatograph paper. A metallic screen prevented direct mosquito contact with the treated paper. Twenty non-blood-fed females (aged 4 to 7 days old) were introduced in the treated chamber and after a 30-se acclimation period, the butterfly valve that separated the two chambers was opened for 10 min. At the end of the test, the butterfly valve was closed and the number of insects in each chamber was recorded. Mosquitoes moving from the treated chamber to the untreated chamber were recorded as ‘escaped’. Conversely, mosquitoes remaining in the treated chamber were considered to have ‘stayed’. Tests were replicated three times for each chemical.

### Contact irritancy assay

These assays were performed using the system described above for the spatial repellent assay, and consisted of two connected tubes used in the WHO test kit and a possible mosquito contact with the chemical. Ten non-blood-fed females (age 4 to 7 days old) were introduced in the treated chamber and each test was performed six times for each chemical. After a 30-s acclimation period, the guillotine valve that separated the two chambers was opened for 10 min allowing the mosquitoes to move freely throughout the arena. Once the guillotine valve was closed, the number of mosquitoes in each tube (‘stayed’ *vs* ‘escaped’) was recorded.

### Toxicity assays

Toxicity assays were performed using a WHO test kit [14]. Twenty non-blood-fed females (aged 4 to 7 days old) were exposed for 1 h to a treated paper (with products or with the solvent only) in the treated tube. Mosquitoes were then transferred to an untreated tube with 10 % honey solution and maintained at 27 °C and 80 % RH. The number of dead and alive *An. gambiae* were recorded after 24 h post-exposure. Each test was replicated three times for each chemical.

We used the same method to analyse the proportion of dead mosquitoes in toxicity assays and the proportion of escaped mosquitoes in both spatial repellency and contact irritancy assays. Data analysis was carried out using the R 2.12.2 software. Tests of treatment effects for the different behavioural assays were carried out on the proportion of escaped or dead mosquitoes in (i) control and treated assays, (ii) essential oil and their associated compounds treated assays. We used Fisher’s exact test corrected according to Bonferroni using the Holm’s sequential method [18]. The behavioural and mortality data were corrected using Sun-Shepard’s formula [19]. For all products and concentrations, these corrected proportions were used to perform a principal component analysis (PCA). Then, a hierarchical ascendant classification (HAC) based on Ward’s algorithm was used to group the compounds of essential oils based on the similarity of their effects using PCA-axes coordinates. This process yielded a binary segmentation tree, reflecting the hierarchy of similarities between responses to plant extracts. The optimal number of classes in the tree was determined by the decrease of the interclass variance.

### Residual efficacy assays

To determine if carvacrol, geraniol, cuminaldehyde and cinnamaldehyde would be efficacious during the 8 h duration of the tunnel experiment, these compounds were tested for their residual toxicity on bednets. Using the same model of the contact irritancy assay, these assays were performed with two connected tubes used in the WHO test kit which allows for possible mosquito contact with the chemical. Instead of using treated paper, treated bednets with products or with solvent only (controls) were rolled around the inner surface of the treated chamber, whereas the inner surface of the untreated chamber was covered by a net, which were treated with neither product nor solvent. Non-treated chromatograph paper were introduced between the bednet and the tube to obtain the same luminosity than for the previous assays. Ten non-blood-fed females (aged 4 to 7 d old) were introduced in the treated chamber at intervals of 0, 3, 6, and 9 h after the treated bednet had been dried. Controls (solvent treated net) were performed at 0 and at 9 h. After a 30-s acclimation period, the slide unit that separated the two chambers was opened for 10 min. Mosquitoes were allowed to move freely and partition in the tubes. Once the slide unit was closed, the number of mosquitoes that ‘escaped’ and ‘stayed’ were recorded as well as their status: alive, knocked-down, or killed. To differentiate between knocked-down *vs* killed insects, the non-mobile mosquitoes were transferred into an untreated tube with 10 % sucrose solution and maintained at 27 °C and 80 % RH. The number of dead and knocked-down i.e. ‘mobile’ *An. gambiae* was recorded after 24 h. Each test was replicated six times for each chemical. Between trials, the chambers were placed in a fume hood to remove the previous treatments and avoid contamination between replicates of the same product and to avoid contamination from one product to the next.

The same method was used to analyse the proportion of repelled, knocked-down, and killed mosquitoes in the assay. The proportions of mosquitoes of each treated assays: 0, 3, 6, and 9 h were compared using Fisher’s exact test to the proportions of the control: at 0 h and 9 h. We tested 9 h because the tunnel test lasts 8 h but other technologies to decrease their volatibility will be necessary to use essential oils or their compounds as bednet treatment. To account for multiple testing, *P*-values of those tests were corrected according to Bonferroni using the Holm’s sequential method. Generalized linear models (GLM) were fitted to assess the effect of time, i.e. persistence effect of the product, on the proportions of repelled, knocked-down, or killed mosquitoes using a binomial distribution with a logit-link function [20]. To assess the adequacy of the models, residuals were checked graphically using a normal quantile-quantile plot.

### Tunnel assays

The tunnel assay system consisted of a square glass tunnel (height 25 cm, width 25 cm, length 60 cm) with netted cage ends (25 cm × 25 cm × 25 cm), subdivided by a changeable piece of netting with 9 × 1 cm holes inserted on a cardboard frame across the tunnel [21].

At one end of the tunnel (bait chamber), a guinea pig was used as bait. The animal was held in a small metallic cage to prevent contact with the netting. At the other end of the tunnel, 100 unfed female mosquitoes (aged 7–9 days old) were introduced at 0900 h local time and the apparatus was left in a dark room maintained at 28 °C and 80 % relative humidity. At 1700 h local time, the numbers of mosquitoes in both compartments were counted and their mortality and blood feeding rates were scored. Tests were replicated two times for each chemical. All the test respected the ethical considerations.

We used the same method to analyse the proportion of mosquitoes that passed through the net, which were blood fed, and killed in the assay. The mosquito rates in control and treated tunnels were compared using Fisher’s exact test. To take into account multiple testing, *P*-values of those tests were corrected according to Bonferroni using the Holm’s sequential method [18].

## Results

### Electrophysiology

**Fig. 1:**
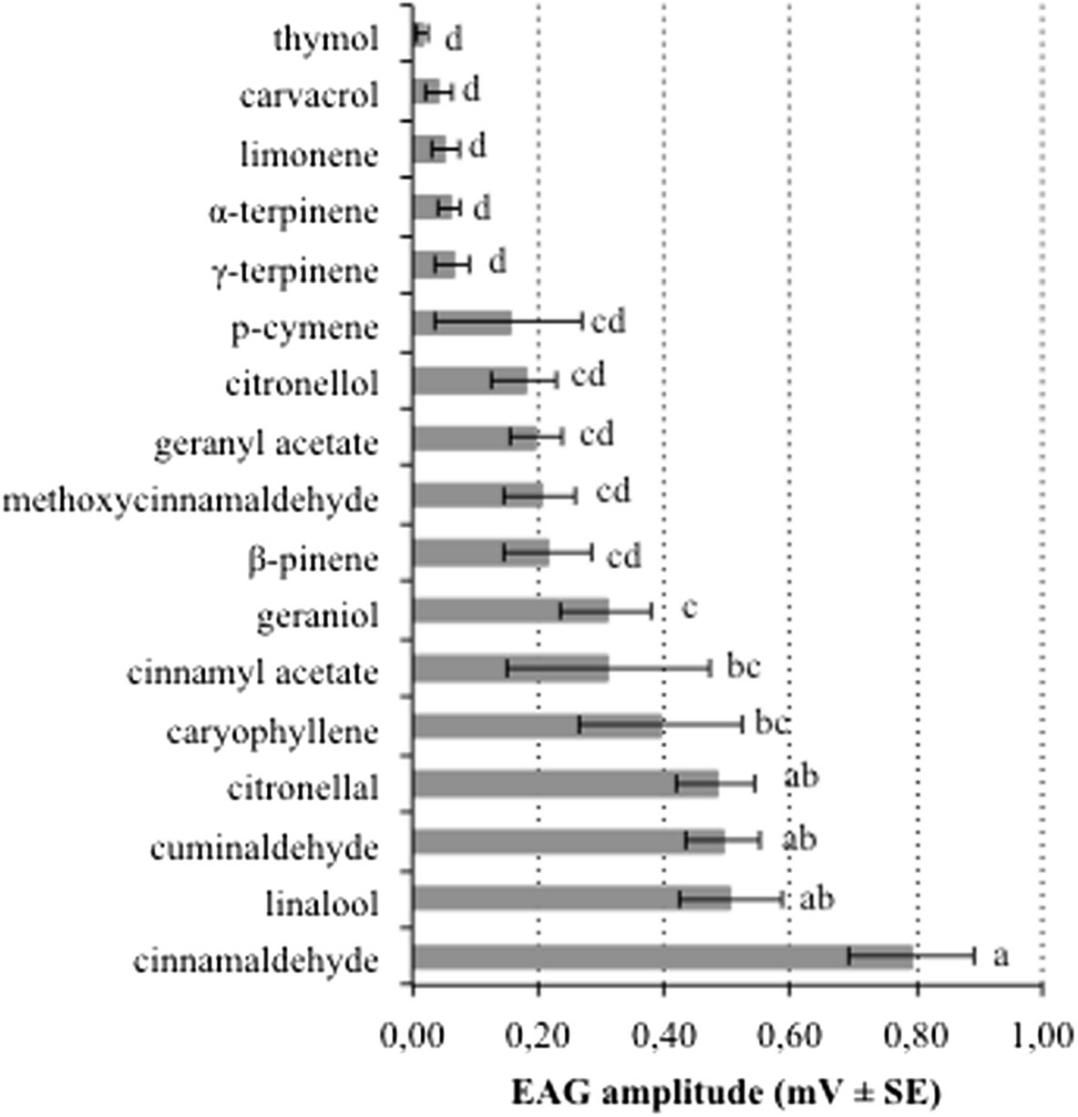
EAG response of *Anopheles gambiae* to 17 synthetic compounds of four essential oils. EAG amplitudes (mean ± SE) are control-adjusted and presented as relative response to the standard, 100 μM octanal. Each compound was tested on 28 female mosquitoes at 1 % (v/v) concentration in ethanol

EAG responses of *An. gambiae* females clearly revealed that the insects responded to the compounds tested (F = 23.5, DF = 18, P < 0.001) (Fig. 1). The strongest responses were elicited by the two aromatic benzaldehydes: cinnamaldehyde and cuminaldehyde, an acyclic monoterpene alcohol (linalool), and an acyclic monoterpene aldehyde (citronellal). Mosquitoes were least responsive to the two cyclic monoterpene phenols tested (carvacrol and thymol). Other compounds elicited intermediate responses (Fig. 1, Appendix: Table 4).

### Repellent assays

**Fig. 2:**
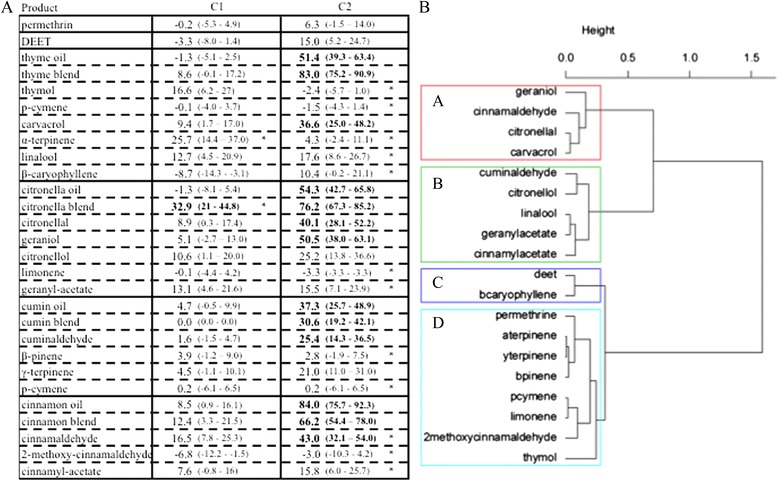
Repellent effect DEET, permethrin and four essential oils and their compounds on *Anopheles gambiae*. Response of 4–7-day-old, non-blood-fed, sugar-fed, Kisumu strain of female mosquitos at two different concentrations (C1 and C2 μl/cm^2^ of product on chromatographic papers, refer to Table 1): **a**. corrected proportion escaping using Sun-Shepard’s formula (confidence interval calculated with the Wald method) by treatment concentration and **b**. dendrogram determined by hierarchical ascendant classification. 1) Pairwise comparison of proportion was done using Fisher’s test. Values in bold lettering were significantly different from the control with the Holm’s sequential Bonferroni correction method. *Pairwise comparison of proportion was done using Fisher’s test between one compound and the essential that it comes from. Values followed by a star were significantly different from the original essential oil with the Holm’s sequential Bonferroni correction method

DEET and permethrin did not exhibit a repellent effect regardless of the concentration tested (C1 and C2) compared to the control (Fig. 2). On the contrary, the essential oils and the blends of the major compounds had a significant repellent effect at the high dose (C2) and at the low dose (C1), only the citronella blend was repellent. The blends were as repellent as their associated essential oils. The following products exhibited the same repellency as the essential oil from which they come from: carvacrol, citronellal, geraniol, citronellol, cuminaldehyde, γ-terpinene. Although cinnamaldehyde was repellent, it was not as repellent as the cinnamon essential oil. According to the similarity of the behavioural response, the clustering procedure based on HAC yielded four contrasted response classes: Class A comprised four products (carvacrol, citronellal, geraniol, and cinnamaldehyde) that were efficacious at C2 and were the most repellent: 36 to 50 % of escaped mosquitoes; Class B was not as repellent and included five products (cuminaldehyde, citronellol, linalool, geranyl acetate, cinnamyl acetate); Class C contained two products (DEET, β-caryophyllene) that were not repellent at any tested concentration even if the highest tested concentration showed a higher repellency effect; Class D consisted of eight products that were not repellent, irrespective of their concentration.

### Irritant assays

**Fig. 3:**
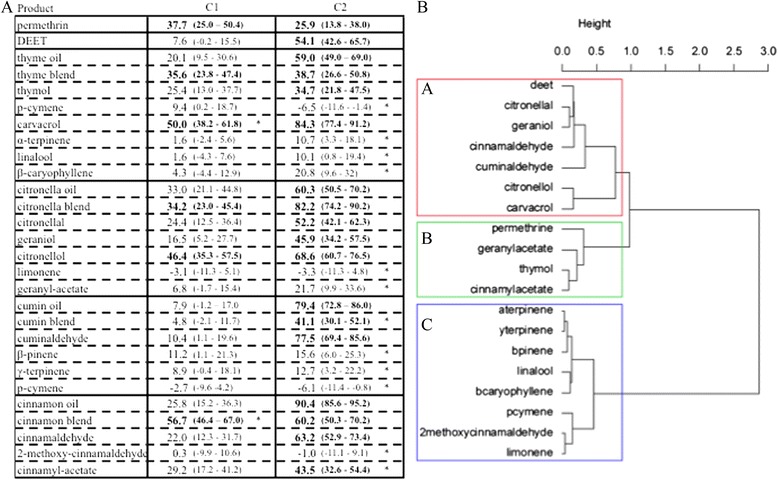
Irritant effect DEET, permethrin and four essential oils and their compounds on *Anopheles gambiae*. Response of 4–7-day-old, non-blood-fed, sugar-fed, Kisumu strain of female mosquitos at two different concentrations (C1 and C2 μl/cm^2^ of product on chromatographic papers, refer to Table 1): **a**. corrected proportion escaping using Sun-Shepard’s formula (confidence interval calculated with the Wald method) by treatment concentration and **b**. dendrogram determined by hierarchical ascendant classification. 1) Pairwise comparison of proportion was done using Fisher’s test. Values in bold lettering were significantly different from the control with the Holm’s sequential Bonferroni correction method. *Pairwise comparison of proportion was done using Fisher’s test between one compound and the essential that it comes from. Values followed by a star were significantly different from the original essential oil with the Holm’s sequential Bonferroni correction method

DEET was a significant irritant at the high dose (C2) but not at the low dose (C1) (Fig. 3). Permethrin was irritant at C1 and C2 concentrations. When permethrin was tested at the C2 concentration, 28.8 % of the mosquitoes were knocked down, and therefore did not escape. All essential oils and blends produced an irritant effect at C2 as did all blends except for cumin blend at C1. At C2 there was no significant difference between the thyme, citronella and cinnamon essential oils and the associated blends. At least one concentration of thymol, carvacrol, citronellal, geraniol, citronellol, cuminaldehyde, cinnamaldehyde, and cinnamyl acetate were irritant compared to the control, and except for cinnamyl acetate, these compounds were as or more irritant as the essential oil that they come from. The HAC could be summarized by three response classes: class A with highly irritant products: 46 to 84 % of escaped mosquito at C2 (DEET, citronellal, geraniol, cinnamaldehyde, cuminaldehyde, citronellol, carvacrol); class B with low irritant products: 21 to 43 % of escaped mosquitoes at C2 (permethrin, geranyl acetate, thymol, cinnamyl acetate) and class C (eight products) with no irritant product.

### Toxicity assays

**Fig. 4:**
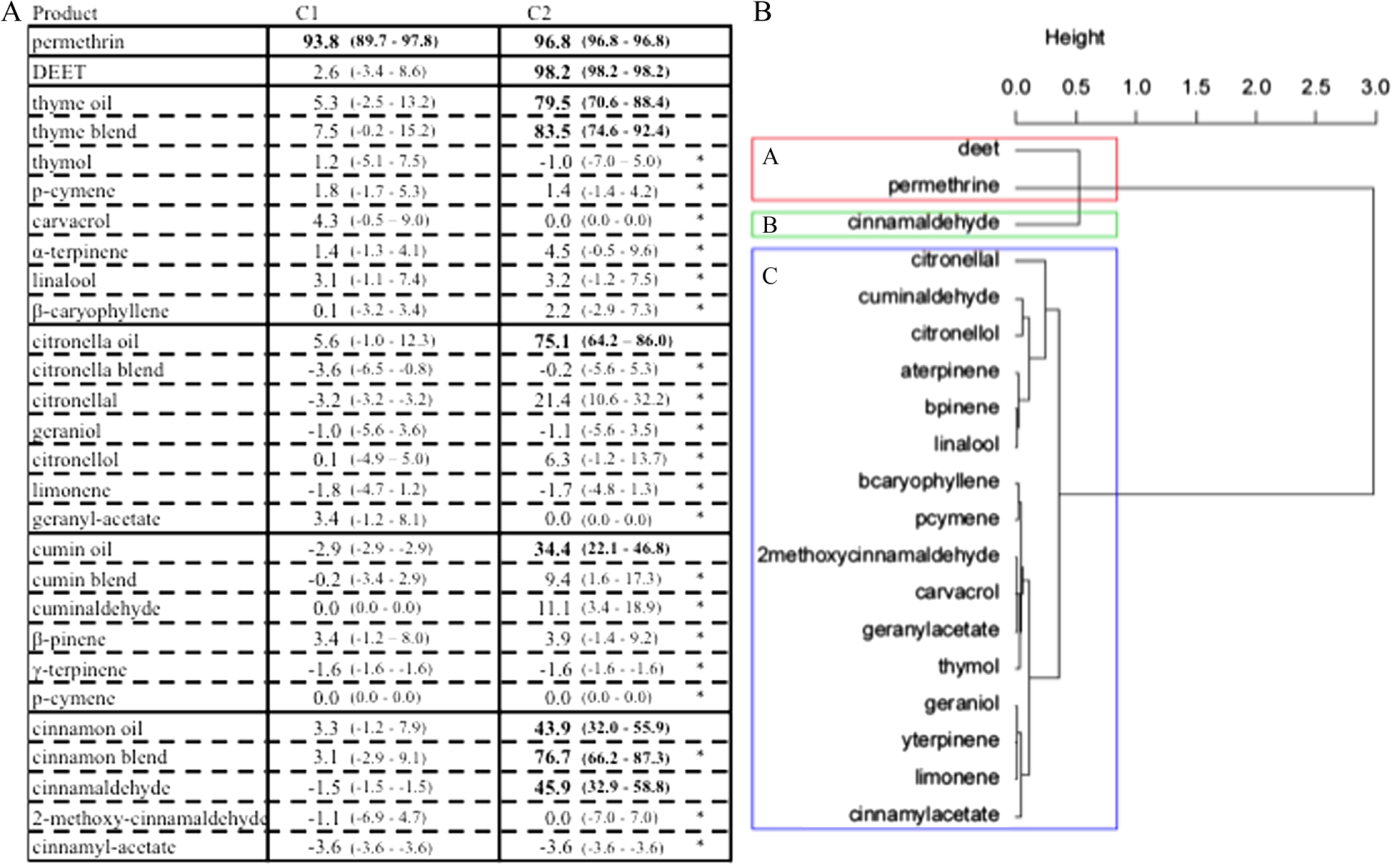
Toxic effect DEET, permethrin and four essential oils and their compounds on *Anopheles gambiae*. Response of 4–7-day-old, non-blood-fed, sugar-fed, Kisumu strain of female mosquitos at two different concentrations (C1 and C2 μl/cm^2^ of product on chromatographic papers, refer to Table 1): **a**. corrected proportion escaping using Sun-Shepard’s formula (confidence interval calculated with the Wald method) by treatment concentration and **b**. dendrogram determined by hierarchical ascendant classification. 1) Pairwise comparison of proportion was done using Fisher’s test. Values in bold lettering were significantly different from the control with the Holm’s sequential Bonferroni correction method. *Pairwise comparison of proportion was done using Fisher’s test between one compound and the essential that it comes from. Values followed by a star were significantly different from the original essential oil with the Holm’s sequential Bonferroni correction method

Permethrin was lethal at the two concentrations tested (C1 and C2) and DEET only at the high dose C2 (Fig. 4). All the essential oils were toxic at C2 but only the thyme blend and the cinnamon blend were toxic at C2 and as or more toxic than their associated essential oils. Only one compound, cinnamaldehyde was toxic at C2 as the *Cinnamomum zeylanicum* essential oil from which it is derived. The HAC analysis yielded three response classes: Class A with two products corresponding to permethrin and DEET; Class B with one product, cinnamaldehyde, the only natural compound that were toxic: 46 % mortality; and Class C with 16 products that were not toxic at all to mosquitoes at the high concentrations tested (C2).

### Residual efficacy assays

The irritant effect of geraniol persisted up to 9 h post bednet treatment: 35 to 45 % escaped mosquitoes (Table 2). However a toxic and knock-down effect was observed at 3 h after the treatment, but not later. The irritant, knock-down and toxic effects of the cinnamaldehyde were still observed 9 h after the treatment, at least 43 % mortality. The irritant effect of the carvacrol was still observed 6 h: 46 % escaped mosquitoes after the treatment, but not later and the knock-down and toxic effect were present after 9 h, with the only observed significant decrease between 0 and 3 h. The irritant effect of cuminaldehyde was still observed 6 h after the treatment: 42 % escaped mosquitoes, but decreased over time. The knock-down and toxic effects were observed just after the treatment but not 3 h later.

### Tunnel assays

At 0.1 μl/cm^2^, the permethrin-treated net reduced significantly the cross rate of mosquitoes through the net less than 60 % (Table 3). There were fewer engorged mosquitoes, maximum 11 % and the mortality rate was significantly higher compared to the control, at least 64 %. At the lowest concentration tested (0.05 μl/cm^2^), cuminaldehyde showed the same effects, but to a lesser extent. At the lowest concentration tested, cinnamaldehyde and carvacrol did not reduce significantly the rate of mosquitoes that passed through the net. However, there were fewer engorged mosquitoes and the mortality rates were significantly higher compared to the control. However, at higher doses these effects were not observed except for cuminaldehyde, for this compound, the mortality rate increased at least 22 % mortality. We tested the nets impregnated with four essential oils to evaluate if synergism would occur between active compounds. A decrease in engorged mosquitoes and an increase in mortality were observed for cumin oil (30 % cuminaldehyde), cinnamon oil (78 % cinnamaldehyde), but not for thyme oil (14 % carvacrol). The 1:1:1:1 blend of compounds (cinnamaldehyde, cuminaldehyde, carvacrol, geraniol) was evaluated for synergistic/additive effects. This blend reduced significantly the engorged mosquito rate compared to the control but there was no reduction of mosquitoes that passed through the treated net and the mortality rate was low. Surprisingly, more mosquitoes passed through the geraniol-treated net compared to the control, but no significant effect was observed upon the engorged and mortality rates. On the contrary, the engorged rate was significantly reduced with citronella oil (22 % geraniol) compared with the control. No significant effects were observed for linalool.

**Table 3:** Efficacy of impregnated bednets in tunnel cage on Anopheles gambiae^a^ females

	Product	Dose (μl/cm2)	N^b^	Passed through net (%)	Engorged (%)	Mortality (%)
1	Control	0	285	86.0	60.7	5.6
	Permethrin	0.1	362	59.1*	11.3*	64.6*
	Geraniol	0.03	300	95.0*	72.0	10.0
	Cinnamaldehyde	0.08	274	80.3	46.0*	22.3*
2	Control	0	283	86.9	68.9	10.2
	Carvacrol	0.03	219	82.2	52.5*	31.1*
	Cuminaldehyde	0.05	263	57.0*	33.8*	44.9*
3	Control	0	260	96.2	87.7	5.8
	Permethrin	0.1	263	51.0*	8.4*	64.6*
	Cuminaldehyde	0.1	259	96.5	76.4	22.0*
	Cinnamaldehyde	0.1	356	94.7	87.9	6.7
4	Control	0	267	98.1	86.5	8.6
	Geraniol	0.1	257	94.6	78.6	11.3
	Carvacrol	0.1	267	91.4*	83.9	10.5
5	Control	0	231	98.7	80.5	6.9
	Blend^c^	0.1	235	81.3	60.4*	6.0
	Thyme oil	0.1	225	93.8	73.3	15.6
	Cinnamon oil	0.1	266	94.7	65.4*	25.9*
6	Control	0	266	95.5	85	5.6
	Cumin oil	0.1	240	93.8	74.6*	6.7
	Citronella oil	0.1	224	95.1	62.5*	3.6
	Linalol	0.1	272	93.8	77.6	4.0

^a^7- to 9-day-old, non-blood-fed, sugar-fed, Kisumu strain

^b^Number of An. gambiae female tested

^c^Blend of carvacrol, geraniol, cinnamaldehyde, cuminaldehyde (1:1:1:1)

*Significant difference (*P* < 0.05, fisher test with the Holm’s sequential Bonferroni correction method) between values for control and treatment tunnels

## Discussion

This study is one of the first to explore electrophysiological and behavioral responses of *An. gambiae* to essential oils and their constituents. For EAG trials, aldehydes as cinnamaldehyde generally elicited stronger responses than did monoterpenes as limonene or terpinenes. Results from the behavioural trials were not always consistent with EAG responses. For example, the EAG response to carvacrol was relatively weak, but strong behavioural responses to this compound were observed. Conversely, mosquitoes exhibited relatively strong EAG responses to cuminaldehyde and linalool, but were not repelled well by these compounds. Correlation was observed for cinnamaldehyde and citronellal, both of which elicited relatively strong EAG and behavioral responses. These results may suggest involvement of different sensory neurons or another pathway than antennal reception in the phenomena of how repellents function. Inconsistencies between electrophysiological and behavioral results have been previously reported [22, 23], and underscore the value of using a variety of research approaches when studying complex behavior such as repellency at the level of the whole organism. In their study on *Aedes aegypti*, Dekker *et al.* [24] examined the repellent effect of electroantennographic detection (EAD)-active compounds of the headspace extracts of crushed *Ocimum forskolei*. They discovered that not all of the EAD-active compounds of this plant were repellent and the repellent compounds were structurally dissimilar. They did not study the repellent effect of the other non-active compounds. In another repellents study, the effect of *Osmanthus fragrans* on the cabbage butterfly *Pieris Rapae*, Ômura *et al.* [25] demonstrated the repellency of γ-decalactone, and this correlated well with its deterrent effect on proboscis extension reflex but not necessarily with antennal sensitivity.

We demonstrated that geraniol, cuminaldehyde, carvacrol and cinnamaldehyde produced consistent behavioural effects compared to those of the essential oils from which they were derived, despite the fact that several were not a major constituent in the respective essential oil. In the repellency assays, the major compound blends were not significantly less repellent than the associated essential oils, suggesting that the major compounds in the blend could be the main essential oils responsible for the observed repellency of the oil. The repellency of carvacrol, citronellal, geraniol, or cuminaldehyde were not significantly different than the repellency of their corresponding essential oils. Therefore, these compounds appeared to be responsible for the repellent effect of their essential oils. Cinnamaldehyde was less repellent than the cinnamon oil suggesting a synergistic or additive effect with one or more other compounds within the oil. The irritant effects of the thyme oil, citronella oil, cumin oil, and cinnamon oil could be explained by thymol and/or carvacrol; citronellal, geraniol, and/or citronellol; cuminaldehyde; and cinnamaldehyde, respectively, because there was no significant difference in irritancy between the single compounds and the associated essential oils. For cumin oil it is possible that there was an antagonistic effect between cuminaldehyde and another constituent because the cumin blend was less irritant than the cumin essential oil. Moreover, none of the major compounds appeared to be responsible for the toxicity observed from the citronella and cumin oil. No difference was observed between cinnamon oil and the cinnamon blend, between cinnamon oil and cinnamaldehyde or between thyme blend and the thyme oil; therefore, the cinnamon oil toxicity could be due to the cinnamaldehyde and the thyme oil toxicity to the major compounds in the blend. Overall, it appeared that the effect of an active compound could be enhanced by other major compounds and/or modulated by minor compounds to give additive or synergistic effects. For example, repellency and toxicity of cinnamaldehyde could be synergised by minor compounds, while the irritancy of carvacrol appeared to be reduced by minor compounds. Repellent and irritant effects of essential oils were usually due to one compound except for citronella oil (citronellal, geraniol and citronellol). So another phenomenon that was identified was the importance of minor compounds in the toxicity of an essential oil. When the minor compounds of citronella and cumin essential oil were not present, toxicity was reduced. This suggests different modes of action for irritancy and repellency than for toxicity. The toxicity of the two other essential oils could be due to a minor compound or a synergistic effect due to the mixture of several compounds. Until now it was assumed that the major compounds of an essential oil reflected the biological response of this essential oil and the response level depended on the concentration of the compound [8]. In previous studies, only the effect of a complete essential oil and sometimes the major compound were studied [26–28]. However, our results showed that the bioactivity of an essential oil does not necessarily mirror the activity of the major component. Instead, activity of an essential oil is complex and depends on interactions between individual compounds and the insect under study.

After 9 h, the efficacy of natural compounds, i.e. their repellency, irritancy, and toxicity, was decreased. The toxic and knock-down effect of all products decreased over time. Geraniol and carvacrol produced relatively stable irritancy, while cuminaldehyde decreased over time, and the irritancy of cinnamaldehyde increased (Appendix: Table 5). However, for cinnamaldehyde and carvacrol, the majority of knocked-down mosquitoes (cinnamaldehyde at 0 h 11.3 % *vs* 0.0 %; carvacrol at 3 h 14.9 % *vs* 3.0 %) were in the non-treated chamber, whereas the majority of dead mosquitoes (cinnamaldehyde at 0 h 64.5 % *vs* 17.7 %; carvacrol at 3 h 50.7 % *vs* 13.4 %) were found in the treated chamber. Therefore, if we take into account the living mosquitoes that ‘stayed’ and ‘escaped’, the irritant effect of geraniol, carvacrol, and cuminaldehyde decreased over time, whereas the repellent effect of cinnamaldehyde increased over time (Appendix: Table 5). The effect of compounds were higher than in the contact irritancy assay while the amount of product per unit area of bednet was the same. Indeed, a net is mainly composed of empty spaces between the polyester fibres where the compound is concentrated and where the tarsal contact occurred. Short duration of protection time is a drawback to essential oils [29]. However, with the new technologies currently available it is possible to increase their residual efficacy. The active products can be encapsulated, used with polymer resins or synergised by other compound like vanillin [27, 30]. However, this rapid decrease of efficacy cannot explain the lack of efficiency of the compound in tunnel tests. For most compounds, repellency appeared to be weaker than the attraction to the host. Cuminaldehyde and cinnamaldehyde, which were the most efficient against *An. gambiae*, were also the most efficient in the tunnel test but less than permethrin. A disadvantage to the use of antifeedant products is that insects can lose sensitivity or change their mode of feeding after repeated and prolonged exposure [31, 32]. This could also be true for repellent/irritant compounds, so the lack of efficiency in time could be due to habituation, not volatility. After a long exposure time, mosquitoes are acclimated to the product and the sensory stimuli have a decreased effect on the nervous system leading to a modified behavioural response [33].

In view of our results, the use of natural compounds to treat nets holds promise, but their use in personal protection should also be considered. The World Health Organisation [3] provided the following definition: «for a material to be valuable as a mosquito repellent it must effectively discourage insect attack on the treated area for many hours and on many different types of surfaces, it must work in different environmental conductions, it must be environmental friendly when applied to human or animal skin, it must be cosmetically acceptable having a pleasant odour, taste and feel, it should also be harmless to clothing, it should have a relatively low cost and be effective against other common types of insects, such as flies». Carvacrol, α-terpinene, citronellal, citronellol, geraniol, cuminaldehyde, cinnamaldehyde and cinnamyl acetate were repellent and/or irritant to insect attack and so they are good candidates for personal protection. However, to be useful, skin repellents need to be innocuous (low toxicity to humans) and to provide protection at least 4 h [34]. Efficacy of essential oils is usually less than 20 min, and moreover, they can be photosensitive and allergenic even if mammalian toxicity is low [26, 35]. In this study we focused on characterization of the bioactive compounds in essential oils and so it is easier to move research forward and determine the characteristics and the target of the active compounds. For example, in cinnamon essential oil the active compound is mainly cinnamaldehyde but Smith Pease *et al.* [36] showed it is allergenic and thus cannot be used as skin repellent. Compounds from citronella can be potential alternatives to repellents, especially since they are non-toxic individually and when mixed. It would be interesting to mix two or three compounds with different effects to avoid habituation behaviour from mosquitoes. The efficacy of the citronella major compounds mixed equals the one of the essential oil so the blend: citronellal-citronellol-geraniol could be interesting. Different mode(s) of action could delay resistance by mutation or insensitivity to one particular product, which has a specific target.

## Conclusions

In our behavioral and toxicity studies, we showed the activity of essential oils did not always correlate with activity expected from the major components. But several of the single compounds of the tested essential oils did exhibit repellency, irritancy or toxicity in *An. Gambiae*. So the biological activity of essential oils is complex due to interactions as additive or synergetic effects between their individual compounds and the insect under study. In our work EAG responses did not correlate consistently with results of behavioral assays. Moreover the data also indicated that the three effects appeared independent, suggesting that repellency mechanism(s) may differ from mechanisms of irritancy and toxicity. Based on the bioassays reported here, some of the compounds merit consideration as alternative bednet treatments, but new technologies have to be used. Moreover, the compounds could be tested on resistant strain to check their efficacy.

## Appendix 1

**Table 4:** EAG response of Anopheles gambiae to 17 synthetic compounds of four essential oils

	Mean^a^	Std dev	Std error	Tukey’s hsd
Cinnamaldehyde	0.79	0.50	0.10	a
Linalool	0.50	0.42	0.08	ab
Cuminaldehyde	0.49	0.30	0.06	ab
Citronellal	0.48	0.33	0.06	ab
Caryophyllene	0.40	0.66	0.13	bc
Cinnamyl acetate	0.31	0.82	0.16	bc
Geraniol	0.31	0.36	0.07	c
β-pinene	0.21	0.36	0.07	cd
Methoxycinnamaldehyde	0.20	0.29	0.06	cd
Geranyl acetate	0.20	0.21	0.04	cd
Citronellol	0.18	0.26	0.05	cd
p-cymene	0.15	0.59	0.12	cd
γ-terpinene	0.06	0.13	0.03	d
α-terpinene	0.06	0.09	0.02	d
Limonene	0.05	0.11	0.02	d
Carvacrol	0.04	0.10	0.02	d
Thymol	0.01	0.05	0.01	d

^a^EAG amplitudes (mean) are control-adjusted and presented as relative response to the standard, 100 μM octanal. Each compound was tested on 28 female mosquitoes at 1 % (v/v) concentration in ethanol

## Appendix 2

**Table 5:** Residual efficacy of the net treatment on Anopheles gambiae^a^

Product^b^	Time (h)	n	Alive			Knocked-down		Killed	
			T	NT	Irritated^c^	T	NT	T	NT
Control	0	66	92.4	6.1	6.2	0.0	0.0	1.5	0.0
Geraniol	0	61	26.2	45.9	63.7	11.5	0.0	16.4	0.0
Geraniol	3	65	46.2	38.5	45.5	3.1	0.0	12.3	0.0
Geraniol	6	66	62.1	34.8	35.9	3.0	0.0	0.0	0.0
Geraniol	9	72	52.8	30.6	36.7	0.0	0.0	0.0	0.0
Control	9	65	90.8	7.7	7.8	0.0	0.0	1.5	0.0
*p*-value (model estimate)^d^				0.005 (−0.1)					
Control	0	62	90.3	9.7	9.7	0.0	0.0	0	0.0
Cinnamaldehyde	0	62	0.0	6.5	100.0	0.0	11.3	64.5	17.7
Cinnamaldehyde	3	66	10.6	15.2	58.9	0.0	6.1	48.5	19.7
Cinnamaldehyde	6	61	1.6	18	91.8	1.6	18.0	42.6	18.0
Cinnamaldehyde	9	66	0.0	22.7	100.0	1.5	12.1	30.3	18.2
Control	9	62	87.1	9.7	10	0.0	0.0	3.2	0.0
*p*-value (model estimate)				0.050 (0.3)					
Control	0	63	92.1	6.3	6.4	0.0	0.0	1.6	0.0
Carvacrol	0	61	1.6	1.6	50.0	0.0	9.8	83.6	3.3
Carvacrol	3	67	3.0	14.9	83.2	3.0	14.9	50.7	13.4
Carvacrol	6	65	6.2	10.8	63.5	9.2	30.8	38.5	4.6
Carvacrol	9	64	15.6	9.4	37.6	15.6	3.1	35.9	4.7
control	9	71	78.9	18.3	18.8	0.0	0.0	2.8	0
*p*-value (model estimate)				0.050 (−0.3)					
Control	0	65	92.3	6.2	6.3	0.0	0.0	0.0	1.5
Cuminaldehyde	0	67	14.9	23.9	61.6	14.9	7.5	17.9	20.9
Cuminaldehyde	3	59	33.9	61.0	64.3	0.0	0.0	5.1	0.0
Cuminaldehyde	6	71	49.3	38.0	43.5	0.0	0.0	8.5	4.2
Cuminaldehyde	9	74	62.2	21.6	25.8	0.0	0.0	1.4	0.0
Control	9	64	89.1	10.9	10.9	0.0	0.0	0.0	0.0
*p*-value (model estimate)				<0.001 (-0.2)					

^a^Proportion of 4- to 7-day-old, non-blood-fed, sugar-fed, females Kisumu strain that were alive, knocked down, and killed by the four tested products after 0, 3, 6 and 9 h of the net treatment in treated and non treated chambers

^b^geraniol (0.023 μl/cm^2^), cinnamaldehyde (0.079 μl/cm^2^), carvacrol (0.014 μl/cm^2^) and cuminaldehyde (0.030 μl/cm^2^)

^c^proportion of alive escaped mosquito

^d^P-value and model estimate of the generalized linear model of the time on the mosquito repellency
